# Prevalence of chronic kidney disease among people living with HIV/AIDS in Burundi: a cross-sectional study

**DOI:** 10.1186/1471-2369-12-40

**Published:** 2011-08-24

**Authors:** Johann Cailhol, Béatrice Nkurunziza, Hassan Izzedine, Emmanuel Nindagiye, Laurence Munyana, Evelyne Baramperanye, Janvière Nzorijana, Désiré Sakubu, Théodore Niyongabo, Olivier Bouchaud

**Affiliations:** 1 Infectious and tropical diseases department, Avicenne Hospital-APHP and Paris 13 University, 125 Route de Stalingrad, 93009, Bobigny, France; 2 Department of Research, National Centre for HIV Reference, Bujumbura, Burundi; 3 Kidney disease department, Pitié-Salpétrière University Hospital, 47-83 Bd de l'Hôpital, 75013, Paris, France; 4 HIV-clinic (CPAMP-CHUK), Bujumbura University Hospital, Burundi; 5 Department of Medicine, Association Nationale de Soutien aux Séropositifs (ANSS), Bujumbura, Burundi; 6 Department of Medicine, Society of Women Against AIDS (SWAA), Bujumbura, Burundi; 7 Department of Medicine, Nouvelle Espérance, Bujumbura, Burundi

**Keywords:** chronic kidney disease, kidney damage, aseptic leukocyturia, proteinuria, risk factors, prevalence

## Abstract

**Background:**

Since little is known about chronic kidney disease (CKD) among people living with HIV/AIDS (PLWHA) in Sub-Saharan Africa, the prevalence and nature of CKD were assessed in Burundi through a multicenter cross-sectional study.

**Methods:**

Patients underwent assessments at baseline and 3 months later. Glomerular Filtration Rate (GFR) was estimated using abbreviated 4-variable Modification of Diet in Renal Diseases (MDRD) and Cockroft-Gault estimation methods. Patients were classified at month 3 into various CKD stages using the National Kidney Foundation (NKF) definition, which combines GFR and urinary abnormalities. Risk factors for presence of proteinuria (PRO) and aseptic leukocyturia (LEU) were further analyzed using multiple logistic regression.

**Results:**

Median age of the patients in the study (N = 300) was 40 years, 70.3% were female and 71.7% were on highly active antiretroviral therapy. Using the MDRD method, CKD prevalence in patients was 45.7%, 30.2% of whom being classified as stage 1 according to the NKF classification, 13.5% as stage 2 and 2% as stage 3. No patient was classified as stage 4 or 5. Among CKD patients with urinary abnormality, PRO accounted for 6.1% and LEU for 18.4%. Significant associations were found between LEU and non-steroidal anti-inflammatory drug (NSAID) use, previous history of tuberculosis, low body mass index and female gender and between PRO and high viral load.

**Conclusion:**

Our study, using a very sensitive definition for CKD evaluation, suggests a potentially high prevalence of CKD among PLWHA in Burundi. Patients should be regularly monitored and preventative measures implemented, such as monitoring NSAID use and adjustment of drug dosages according to body weight. Urine dipsticks could be used as a screening tool to detect patients at risk of renal impairment.

## Background

Since the era of highly active antiretroviral therapy (HAART), mortality and morbidity among HIV-infected patients in high-income countries have dramatically shifted from opportunistic infections (OI) to chronic conditions [1]. Since 1995 in the United States, chronic kidney diseases (CKD) have become the fourth leading cause of mortality among HIV-infected patients [2].

In resource-limited countries, while OI still account for the majority of HIV-related mortality and morbidity, their incidence is likely to decline, paralleling the continued implementation of earlier and wider access to HAART. OI will be replaced by chronic diseases, including CKD, as a consequence of longer survival [3]. Patients from Sub-Saharan Africa (SSA) are of particular concern, since they accumulate CKD risk factors both related to HIV (late HIV diagnosis, OI, nephrotoxic OI- or HIV-related drugs) and non-related (hypertension, diabetes and ethnicity) [4-7].

Data on PLWHA renal function in SSA are scarce, despite being the area hardest hit by the pandemic. Data that is available on CKD prevalence in PLWHA ranges from 6% to 45%, although a variety of definitions have been used [7-11].

In Burundi, CKD data are inexistent, both in the general population and among PLWHA. We assessed the prevalence of CKD among PLWHA in Burundi through a multicenter cross-sectional study. We also assessed risk factors related to CKD among PLWHA in order to draw recommendations in terms of monitoring.

## Methods

A multicenter cross-sectional survey was conducted between February 2008 and February 2009 in 4 outpatient HIV-clinics in Bujumbura, Burundi.

All HIV_1 _positive adults above the age of 18 who attended the clinics for routine visits during the survey period were included, until the predefined objective of 300 patients (75 patients per site) was reached. The assumptions underlying the calculation of the sample size were that 250 000 persons were HIV positive in Burundi, at least 12% of whom having CKD, based on the literature reviewed at that time [12-14]. The measurement error was set at +/- 4%, leading to a required 253 patients, to whom we added an extra 20% to account for the expected lost to follow-up percentage, leading to a total of 300 patients.

Patients were assessed at baseline and 3 months later for those identified as possible CKD-patients at baseline. Baseline parameters were as follows: socio-demographic, medical and therapeutic histories; physical assessments including weight, height and blood pressure; blood examinations including CD4 cell count, HIV_1 _viral load, serum creatinine (auto-creatinine Liquicolor^®^, Human, Wiesbaden, Germany; result expressed in μmol/l), hepatitis C and B (HBs antigen) serologies; and urinalysis using a dipstick. At month 3, possible CKD-patients were reassessed through physical examination, serum creatinine measurement and urine dipstick. Upon this reassessment, those classified as CKD-patients underwent further blood examination, consisting of serum levels of sodium, potassium, uric acid, bicarbonates, phosphorus, and patients diagnosed with glycosuria underwent serum glycemia in order to discriminate diabetes mellitus.

Urine samples were assessed for the presence of protein, blood, leucocytes, nitrite and glucose using dipsticks (Multistix 8SG^®^, Bayer Diagnostics, Salisbury, United Kingdom) and results were analyzed by an automatic analyzer (Clinitek Status^®^, Bayer Diagnostics, Salisbury, United Kingdom) to ensure standardization. Proteinuria, leukocyturia, hematuria and glycosuria were measured in a semi-quantitative way (0; 1 + to 3 +), whereas nitrite was simply measured in terms of absence or presence.

The Glomerular Filtration Rate (GFR) was estimated by 2 methods: a) abbreviated 4-variable Modification of Diet in Renal Disease (MDRD) [GFR_MDRD _(ml/min/1.73 m^2^) = 186.3 (serum creatinine/88.4)^-1.154 ^(age^-0.203^) (0.742 if female) (1.212 if of African origin)] [15]; b) Cockroft-Gault (CG) [GFR_CG _(ml/min) = {(140 - age) (weight)(1.23 if male or 1.04 if female)(1.18 if of African origin)}/serum creatinine] [16]. Serum creatinine was measured in μmol/l.

The definition and classification of CKD were based on the National Kidney Foundation (NKF) guidelines [15]. Accordingly, the definition of possible CKD at baseline was: GFR_CG _< 60 ml/min or GFR_MDRD _< 60 ml/min/1.73 m^2 ^and/or kidney damage defined as a urinary abnormality at any GFR level. In addition to proteinuria, which was the most evidence-based marker of kidney damage according to NKF, 2 other markers were included, leukocyturia and hematuria. Urinary abnormality was defined by isolated proteinuria ≥ 1+ or isolated hematuria ≥ 2+ or isolated leukocyturia ≥ 2+ or the association of hematuria ≥ 1+ with leukocyturia ≥ 1+ or proteinuria ≥ 1+. Patients with urine dipstick abnormalities as described above at baseline underwent a urine bacterial examination. If a urinary tract infection was diagnosed, the patient was prescribed an appropriate antibiotic and urine samples were again assessed by dipstick 10 days after the completion of treatment, in order to both confirm the disappearance of the urine infection and reassess urinary abnormalities. If leukocyturia persisted, the patient was screened for symptoms of active tuberculosis.

Patients were classified as CKD if GFR_CG _< 60 ml/min, GFR_MDRD _< 60 ml/min/1.73 m^2^, or if urinary abnormalities persisted at month 3 after the baseline assessment.

In addition, persistent abnormalities as highlighted by urine dipstick at month 3 were categorized into 5 groups (a-e): a) proteinuria (PRO) in case of either isolated proteinuria ≥ 3+ or proteinuria ≥ 1+ associated with hematuria ≥ 2+ or leukocyturia ≥ 2+; b) leukocyturia (LEU) in case of leukocyturia ≥ 2+, isolated or not, without proteinuria; c) hematuria (HEM) in case of isolated hematuria ≥ 2+; d) glycosuria (GLY) in case of normoglycemic glycosuria; e) any other combination of abnormalities (i.e. proteinuria ≥ 1+ but < 3+, isolated or associated with hematuria or leukocyturia = 1+) was classified as "other".

Due to local constraints, it was not possible to perform ultrasonographic examination or renal biopsy in patients diagnosed with CKD.

Stata version 8 (Stata corporation, College Station, Texas, USA) was used for all statistical analyses. We provided descriptive analysis of the study population and GFR, using the 2 methods of measure, CG and MDRD. Prevalences of CKD were calculated together with their 95% confidence intervals, after checking the normality of their distribution. Lost to follow-up patients were considered as either being all affected by CKD or none being affected, to provide upper and lower estimates of CKD prevalence as part of the sensitivity analysis. As CKD is a mixture of multiple etiologies, we focused on the analysis of abnormalities as highlighted by the use of urine dipsticks. Patients with glycosuria, related to either pregnancy or diabetes mellitus, were excluded from the risk factor analysis, as were patients with known causes of CKD, namely diabetes and hypertension.

Predictors of PRO and LEU were analyzed, using a univariable analysis and multivariable logistic regression model. Numerical variables were included as continuous variables after verifying their normality, otherwise they were used as categorized variables. Variables associated with PRO or LEU, with a p-value < 0.2 in univariate analysis, were included in a multivariable analysis. The most parsimonious final model was determined by using a stepwise method, while variables were also removed when statistical interactions with a biological explanation were present (e.g. between WHO stage and a history of tuberculosis).

When comparing GFR_MDRD _and GFR_CG_, we adjusted GFR_CG _for the body surface area (BSA), using the Dubois formula [17]: BSA adjusted GFR_CG _= (GFR_CG _× 1.73)/BSA, with BSA (m^2^) = (weight ^0.425 ^× height ^0.725^) × 0.007184 (weight in kilograms, height in centimeters). We then computed Pearson's correlation coefficient and used the Bland-Altman method to compute the agreement between the BSA adjusted GFR_CG _and GFR_MDRD_. The kappa index was used to analyze the level of agreement between the NKF stages classification obtained using GFR_MDRD _and GFR_CG. _Agreement was considered "moderate" when the index values ranged from 0.41 to 0.60, "good" for values ranging from 0.61 to 0.80 and "excellent" for values from 0.81 to 1.

The study was approved by the National Ethics Committee of Burundi. Before inclusion in the study, patients were informed and asked to sign a study consent form in Kirundi, the local language. Abnormal results were forwarded to the physician in charge, such that appropriate decisions could be taken.

## Results

**Figure 1:**
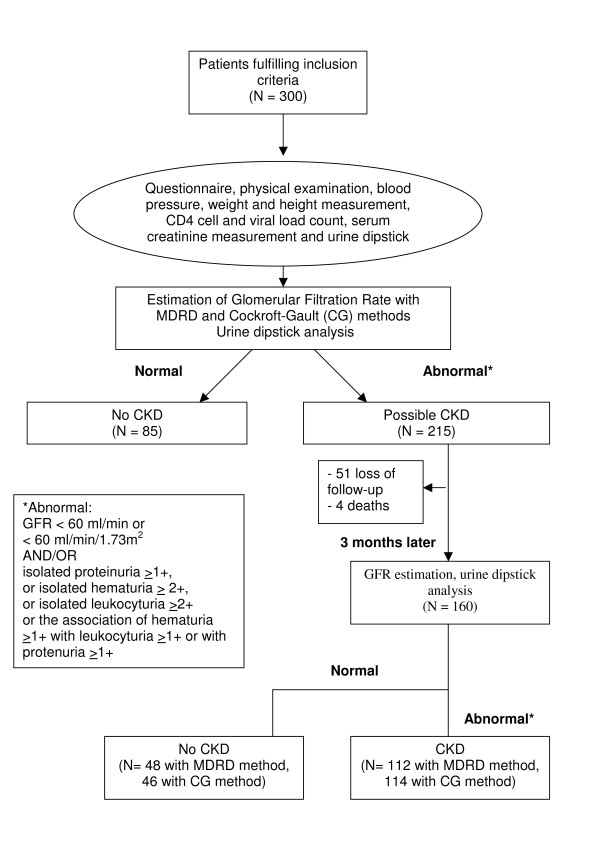
**flowchart of included patients**.

A total of 300 patients were included, 75 patients from each of the 4 HIV-clinics. Patient flow is summarized in Figure 1. Baseline data are provided in Table 1. All patients were African, the median age was 40.1 years old and 70.3% were women. Median CD4 cell count was 325 cells/mm^3 ^(IQR 205-502). Two hundred and five patients (69.8%) were on HAART, with a median duration of 1 year (IQR 1-3) and 47.9% had previously been treated with non-steroidal anti-inflammatory drugs (NSAID). Among those on HAART, 78% were receiving the WHO recommended regimen (consisting of AZT + 3TC + NVP 28.8%, AZT + 3TC + EFV 10.7%, D4T + 3TC + NVP 27.8%, D4T + 3TC + EFV 10.7%) and the remaining 22% were receiving various other drugs, including 5 patients on tenofovir and one on indinavir. At baseline, median GFR_MDRD _was 99.7 ml/minute/1.73m^2 ^and median GFR_CG _was 111 ml/min.

**Table 1:** Descriptive analysis of the 300 included patients at baseline

Variables	Estimates	N	Missing information (N)
Socio-demographic factors			
Age (years), median (IQR)	40.1 (33-46.5)		0
Female, % (95% CI)	70.3 (65-75)	211	0
Body mass index (kg/m^2^), median (IQR)	21.8 (19.3-24.2)		7
HIV-related factors			
CD4 (cells/mm3), median (IQR)	325 (205-502)		14
Viral load (log_10 _copies/ml), median (IQR)	1.65 (< 1.6-3.1)		11
Current use of HAART, % (95% CI)	69.8 (65.5-75.0)	205	3
Current use of cotrimoxazole, % (95% CI)	87 (82-90)	260	0
History of tuberculosis, % (95% CI)	39.2 (33.7-45)	117	2
Kidney risk factors			
History of diabetes, % (95% CI)	2.0 (0.7-4.3)	6	2
History of hypertension*, % (95% CI)	2.7 (1.1-5.2)	8	1
Positive hepatitis B serology, % (95% CI)	5.0 (2.7-8.2)	14	19
Positive Hepatitis C serology, % (95% CI)	5.3 (3.0-8.6)	15	20
History of aminoglycosides use, % (95% CI)	8.7 (5.6-12.8)	23	37
History of NSAID use, % (95% CI)	47.9 (42-54)	135	18
Renal functions			
GFR Cockroft-Gault (ml/min), median (IQR)	99.7 (79-126)		0
GFR MDRD (ml/min/1.73 m^2^), median (IQR)	111 (88-138)		0

IQR, Inter Quartile Range; 95% CI, 95% Confidence Interval; HAART, Highly Active Antiretroviral Therapy; NSAID, Non Steroidal Anti-Inflammatory Drugs; GFR, Glomerular Filtration Rate; MDRD, Modification of Diet in Renal Disease

* TAS > 16 or TAD > 10 at physical assessment or a previous history of hypertension.

Among the 300 patients, 215 were identified at baseline as possible CKD-patients according to the NKF definition and consequently were eligible for the second assessment 3 months later. Eighty-five patients were classified at baseline as non-CKD and did not undergo the month 3 assessment. Out of the 215 possible CKD-patients, 51 were loss to follow-up and 4 patients died leading to 160 possible-CKD patients at the time of the month 3 assessment.

After the month 3 assessment, the prevalence of CKD was 45.7% (N = 112, 95% CI [39.7-51.7]) using the MDRD estimation method and 46.5% (N = 114, 95% CI [40.3-52.7]) using the CG estimation method, out of the final study population of 245 patients. Fourteen patients had urinary tract infections at baseline and received antibiotics. No patient with glycosuria was diagnosed with pregnancy, and no urinary tuberculosis was diagnosed among patients with aseptic leukocyturia. Eight women were pregnant, two patients were taking isoniazid prophylaxis and five patients were using tenofovir.

Patient classification according to the NKF CKD stages is provided in Table 2. Urinary abnormalities with normal GFR were found in 74 patients (30.2%) according to the MDRD estimation and in 65 patients (26.5%) according to the CG estimation (stage 1). Thirty three (13.5%) and 5 (2%) patients were respectively classified at stages 2 and 3 of the NKF classification, using the MDRD estimation. Using the CG estimation, 37 patients (15.1%) were classified at stage 2 and 12 patients (4.9%) at stage 3. No patient was classified at stages 4 or 5.

**Table 2:** Chronic Kidney Disease stages according to the National Kidney Foundation), using Cockroft Gault and Modification of Diet in Renal Disease Glomerular Filtration Rate estimations (N = 245¤)

CKD stage	GFR estimation (ml/min/1.73 m^2 ^if MDRD, ml/min if CG)	MDRD estimation		CG estimation	
		**N**	**%**	**N**	**%**

0	≥ 90, without urine dipstick abnormality	133	54.3	131	53.4
1	≥ 90, with urine dipstick abnormality*	74	30.2	65	26.5
2	60-89, with urine dipstick abnormality*	33	13.5	37	15.1
3	30-59, with or without urine dipstick abnormality*	5	2.0	12	4.9
4	15-29, with or without urine dipstick abnormality*	0	0	0	0
5	< 15, with or without urine dipstick abnormality*	0	0	0	0
Total		245	100	245	100

* urine dipstick abnormalities:

- Proteinuria ≥ 1+,

- Isolated hematuria or leukocyturia ≥ 2+

- Hematuria ≥ 1+ associated with leukocyturia ≥ 1+

CKD, Chronic Kidney Disease; GFR, Glomerular Filtration Rate; MDRD, Modification of Diet in Renal Disease; CG, Cockroft-Gault

¤ 245 assessable patients = 85 non CKD patients at baseline + 160 assessable patients at month 3

Out of the 245 remaining patients, 110 had persistent urinary abnormalities. Abnormalities were classified LEU in 18.4% of cases (95% CI [13.1-23.2], N = 45), PRO in 6.1% (95% CI [3.1-9.1], N = 15), HEM in 3.3% (95% CI [1.0-5.5], N = 8) and "other" in 17.1% (95% CI [12.4-21.9], N = 42). No GLY was detected.

Five patients with CKD from hypertension or diabetes were excluded for the purpose of the risk factor analysis. Results of univariable and multivariable analysis for risk factors of LEU are shown in Tables 3 and 4. Using the univariable analysis, the following factors were all associated with LEU: previous use of aminoglycosides, NSAID and acyclovir, current use of cotrimoxazole and HAART, high viral load, low Body Mass Index (BMI), low CD4 cell count, female gender and a previous history of tuberculosis. Risk factors of LEU, in multivariable analysis, were as follows: female gender (OR = 6, 95% CI [2.0-18.0], p < 0.01), a previous history of tuberculosis (OR = 2.9, 95% CI [1.4-6.0], p < 0.01), a history of NSAID use (OR = 2.2, 95% CI [1.0-4.6], p = 0.03) and low BMI (OR = 0.8 per 0.1 kg/m^2 ^increment, 95% CI [0.8-0.9], p = 0.01). Pregnancy was not associated with LEU.

**Table 3:** Univariable analysis of risk factors for leukocyturia (LEU)

		N	LEU (N)	OR	95% CI	p
Age (1 year increment)				0.9	0.9-1.0	0.59
Gender	Male	63	4	1		
	Female	133	40	4.7	1.6-13.8	< 0.01
History of tuberculosis	No	144	18	1		
	Yes	95	25	2.5	1.3-4.9	< 0.01
Hepatitis B positive	No	215	38	1		
	Yes	12	2	1.0	0.2-5.1	0.93
Hepatitis C positive	No	213	37	1		
	Yes	13	3	0.7	0.2-2.7	0.60
Body Mass index (0.1 kg/m^2 ^increment)				0.9	0.8-0.9	0.03
History of NSAID use	No	116	15	1		
	Yes	110	29	2.4	1.2-4.8	0.01
Current use of Cotrimoxazole	No	53	2	1		
	Yes	181	42	7.7	1.8-33.3	< 0.01
Current use of HAART	No	67	7	1		
	Yes	171	37	2.3	0.9-5.6	0.05
Viral load (log_10 _copies/ml)	< 1.6	122	29	1		
	[1.6-3.3]	59	8	0.5	0.2-1.2	
	[3.3-8]	49	7	0.5	0.2-1.3	0.15
History of aminoglycosides use	No	191	12	1		
	Yes	19	7	2.7	0.9-7.3	0.05
History of acyclovir use	No	162	27			
	Yes	59	17	2.0	1.0-4.1	0.04
WHO stage	1	36	2	1		
	2	48	8	3.7	0.7-18.5	
	3	122	25	4.4	0.9-19.5	
	4	28	9	8.0	1.6-41.1	0.03
CD4 (1 cell/mm^3 ^increment)				0.9	0.9-1.0	0.06

LEU, Leukocyturia; OR, Odds Ratio; 95% CI, 95% Confidence Interval; HAART, Highly Active Antiretroviral Therapy; NSAID, Non Steroidal Anti-Inflammatory Drugs; WHO, World Health Organization;

**Table 4:** Multivariable analysis of risk factors for leukocyturia (N¤ = 221)

		Adjusted OR	95% CI	p
Gender	Male	1		
	Female	6.0	2.0-18.0	< 0.01
History of tuberculosis	No	1		
	Yes	2.9	1.4-6.0	< 0.01
Body Mass index (0.1 kg/m^2 ^increment)		0.8	0.8-0.9	0.01
History of NSAID use	No	1		
	Yes	2.2	1.0-4.6	0.03

OR, Odds Ratio; 95% CI, 95% Confidence Interval; NSAID, Non Steroidal Anti-Inflammatory Drugs;

¤ multivariable analysis with 221 patients without missing data among the 240 assessable patients

Results from the risk factor analysis of PRO are shown in Tables 5 and 6. In the univariable analysis, PRO was significantly associated with the current HAART use and high viral load. In the multivariable analysis, PRO only remained associated with a viral load higher than 3.3 log_10 _copies/ml (OR = 7.7, 95% CI [1.9-30.5], p < 0.01).

**Table 5:** Univariable analysis of risk factors for proteinuria (PRO)

		N	PRO (N)	OR	95% CI	p
Age (1 year increment)				0.9	0.9-1.0	0.12
Gender	Male	67	5	1		
	Female	173	10	0.8	0.2-2.3	0.63
CD4 (1 cell/mm^3 ^increment)				0.9	0.9-1.0	0.44
Hepatitis B positive	No	215	14	1		
	Yes	12	1	0.8	0.1-6.3	0.80
Hepatitis C positive	No	213	14	1		
	Yes	13	1	0.8	0.1-7.0	0.87
Current HAART	No	67	8	1		
	Yes	171	7	0.3	0.1-0.9	0.03
Viral load (log_10 _copies/ml)	< 1.6	122	3	1		
	[1.6-3.3]	59	4	2.9	0.6-13.3	
	[3.3-3.8]	49	8	7.7	1.9-30.6	< 0.01

PRO, Proteinuria; OR, Odds Ratio, 95% CI, 95% Confidence Interval, HAART, Highly Active Antiretroviral Therapy

**Table 6:** Multivariable analysis of risk factors for proteinuria (N¤ = 230)

		Adjusted OR	95% CI	p
Viral load (log_10 _copies/ml)	< 1.6	1		
	[1.6-3.3]	2.9	0.6-13.3	
	[3.3-3.8]	7.7	1.9-30.6	< 0.01

OR, Odds Ratio; 95% CI, 95% Confidence Interval

¤ multivariable analysis with 230 patients without missing data among the 240 assessable patients

The results of the sensitivity analysis were as follows: if loss to follow-up patients were all classified as CKD (according to the MDRD method), or as non-CKD, prevalence of CKD would have been 55.6% and 37% respectively.

The Pearson's correlation coefficient between BSA adjusted GFR_CG _and GFR_MDRD _was 0.91. The Bland Altman method provided a mean difference of -1.7 ml/min/1.73 m^2 ^between BSA adjusted GFR_CG _and GFR_MDRD _[95% CI -3.7 to -0.3]. The kappa index of agreement between the NKF stages classification, based on either GFR_MDRD _or GFR_CG _was 0.79.

## Discussion

The prevalence of CKD among this population of PLWHA in Burundi accounted for nearly half of the sample: 45.7% using the MDRD method and 46.5% using the CG method. LEU was more frequently identified than PRO, accounting for 18.4% and 6.1%, respectively.

This prevalence of CKD is one of the highest ever found among both resource-rich and resource-limited countries [8,9,11,18,19]. It might be related to the use of the new and sensitive, but less specific definition of CKD, incorporating urinary abnormalities [15]. A sensitive but less specific tool might be used as a screening tool to detect patients at risk of developing renal impairment and needing careful renal monitoring. Cross-studies' comparison of CKD prevalence is hampered by the heterogeneity of definitions used, such as chronic kidney failure with a GFR_CG _below 60 ml/min or a combination of definitions assessing the presence of proteinuria together or not with the GFR [8,9,11,18]. The only study using the same definition as that presented here was performed in the USA. In that case a CKD prevalence of 23.7% was reported using a once-off measurement of creatinine [19].

Patients at NKF stage 3 (with GFR_MDRD _< 60 ml/min/1.73 m^2^) represented 2% of our sample, using the MDRD estimation and 4.9% using the CG estimation. Quite similar rates were found in Kenya and Spain, 4.8% [11] and 7.6% [20], respectively. Patients with GFR_MDRD _< 90 ml/min/1.73 m^2 ^represented 15.5% in our sample. Overton et al. reported a much higher prevalence of 43% in the United States, but hypertensive patients were included in their sample and constituted 33% of their population [21]. A study carried out in Uganda showed that the proportion of patients with GFR_CG _< 60 ml/min was 12% at baseline and decreased with HAART to 4%, after 24 months of follow up [10]. This latter rate is similar to our study, where 4.9% of patients had GFR_CG _< 60 ml/min and could be linked to the fact that the majority of our patients (71.7%) were on HAART. Indeed, Kalayjian et *al*. reported significant GFR improvement following HAART prescription, supporting the hypothesis that kidney inflammation is secondary to immune activation [22]. In the DART trial conducted in Uganda and Zimbabwe, investigators reported a moderately decreased median GFR (30-60 ml/min) in 7% of the patients using the CG formula and 3% using the MDRD formula (adjusted for ethnicity), close to the 4.9% and 2% found in our study, even though urinary abnormalities were not taken into account [23].

The presence of LEU could be interpreted as a marker of interstitial nephritis. The association between a history of tuberculosis and LEU (OR = 2.91) possibly reflects sequelae of the urological extension of tuberculosis which is common but underdiagnosed [24]. In our sample, the proportion of patients with a history of tuberculosis was very high (39.2%). Those patients may have had urological extension of tuberculosis and may have had secondarily developed leukocyturia. In Burundi, the availability of tuberculosis diagnosis tools such as mycobacterial culture is limited. The association of LEU and NSAID is not surprising. Interstitial nephritis has been reported after exposure to NSAID with leukocyturia tending to last from a month to a year [25]. The mechanism and risk factors of NSAID toxicity have not yet been clearly identified [25]. NSAID may also aggravate previously damaged kidneys. In South Africa, Fabian et *al *screened 585 HAART-naïve PLWHA for urinary abnormalities, using a once-off dipstick and reported leukocyturia in 30.3% of the patients, 70.9% of whom were sterile [26]. The authors hypothesized that this could be caused by interstitial nephritis, either due to HIV-infection or to drug toxicity.

In our sample, an increase in BMI was negatively associated with the presence of LEU (OR = 0.8 per 0.1 kg/m^2 ^increment). In Burundi, adjusting dosages to body weight is not always possible. Consequently, patients with a low BMI are exposed to a greater concentration of drugs and therefore to acute tubular necrosis, with sequellar interstitial damage (e.g. with aminoglycosides). In our results, the use of aminoglycosides was associated with the presence of LEU in univariable analysis, but due certainly to a lack of power, the association did not remain in the multivariable analysis. Association between low BMI and renal impairment was also reported in Nigeria. This was explained as being due to an advanced renal disease [8]. However, in our sample, the stage of renal impairment was not advanced enough for the association to be interpreted in this way. Hepatitis C has also been associated with proteinuria in the literature [27], but our sample size prevented us from detecting such an association.

The association between PRO and high viral load may reflect the presence of HIV associated nephropathy (HIVAN). Prevalence of HIVAN at the time of the study was estimated at between 3.5% and 12%, the highest rates coming from autopsy-based estimations [28]. However, with the availability of HAART, at least in resource-rich countries, a change in the epidemiology of CKD is being reported. HIVAN is progressively being replaced by other CKD mechanisms, such as arterionephrosclerosis [29] and drug-related toxicity [19]. In our sample, the low prevalence of PRO could be related to the fact that nearly half of the patients had undetectable VL. The genetic susceptibility to HIVAN, through the gene MYH9 may also be different in this part of SSA, as suggested by other studies in East Africa, where the prevalence of HIVAN was low [11,18].

In our sample, both correlation and agreement between GFR_MDRD _and GFR_CG _were good. Buitrago et *al *found a moderate level of agreement (kappa index of 0.55) but focused on agreement for CKD at stage 3, which was underrepresented in our sample [30]. The MDRD method tended to slightly overestimate the GFR compared with the CG method, as suggested by the negative mean difference, though this was not statistically significant. The overestimation of GFR by MDRD compared with CG was also reported in the DART trial. In our population, the proportions of underweight (BMI < 18.5) and obese (BMI > 30) persons were 6% and 17% respectively, similar to those in the DART trial (respectively 5% and 18%), supporting the hypothesis that weight distribution had an influence on the MDRD/CG estimations. We also agree with our colleagues that the use of MDRD and CG formulas require further validation for East Africa, based on comparison with a reference technique of GFR measurement [11,23].

We acknowledge several limitations to our study, due to the challenges of performing specialized examinations in Burundi. The great difficulty in performing ultrasound measurements and renal biopsies prevented precise diagnosis of patients diagnosed with GFR and/or urine dipstick abnormalities. We therefore used urinary abnormality as a tool to categorize patients and discussed possible diagnosis linked to them. Among female patients, chronic pyelonephritis or asymptomatic bacterial urinary tract infections could have been underdiagnosed due to a low quality of bacteriological examination, especially for those on cotrimoxazole. This could have led to an overestimation of CKD prevalence and could partly explain the association between female gender and LEU. Tuberculosis may also have been underdiagnosed, but none of the patients presented with clinical signs of active disease. The presence of leukocyturia and hematuria might also be related to genito-urinary diseases other than tuberculosis or urinary tract infections. Underdiagnosis of these infections might have led to an overestimation of CKD prevalence in our sample. The baseline kidney status of the non-HIV population is largely unknown in Burundi and we cannot attribute kidney function abnormalities to HIV-related factors with certitude. Similarly, the Dubois equation was used although the relevance of this equation in this population is unknown. Despite these biases, we believe the results of this study are important in helping raise awareness of CKD among PLWHA in this context.

## Conclusions

Whereas previous studies mainly focused on HIVAN, there is now a need to shift towards a more comprehensive review of all the potential risk factors associated with CKD. HIV is a chronic disease and patients are now exposed to a range of non HIV-specific kidney risk factors, such as arteriosclerosis, hypertension or diabetes mellitus. In addition, PLWHA are more likely to experience kidney damage, due to a higher susceptibility to renal infections including tuberculosis, to longer and more frequent exposure to multiple drugs, HIV-related or not, and finally due to inflammation. Thus, diagnosing HIV earlier could lead to earlier HAART prescription, as recently recommended by the WHO [31]. This could also help decrease the incidence of tuberculosis and therefore urological sequelae. Further context-specific studies are required as well as comprehensive care for PLWHA, including kidney risk factors assessments and weight-adjusted prescriptions. NSAID use, either upon prescription or over-the-counter, should be closely monitored. The increasing use of tenofovir-based regimens is also of concern, as incomplete reversibility of renal toxicity has been reported, mitigated by other reassuring preliminary results [32-34]. The use of a sensitive but less specific definition of CKD, such as that used in this study, might help to detect patients at risk of both developing a GFR decrease and further renal impairment. In the context of Burundi and most resource-limited countries, the use of a urine dipstick as a screening test may be easily implemented as part of the routine monitoring of PLWHA. Further studies with long-term follow-up are needed to analyze the renal outcome of patients diagnosed as CKD using this sensitive definition.

## List of abbreviations used

BMI: Body Mass Index; BSA: Body Surface Area; CG: Cockroft-Gault; CI: Confidence Interval; CKD: Chronic Kidney Disease; GFR: Glomerular Filtration Rate; GLY: Glycemia; HAART: Highly Active Antiretroviral Therapy; HEM: Hematuria; HIV: Human Immunodeficiency Virus; LEU: Leukocyturia; MDRD: Modification of Diet in Renal Disease; NKF: National Kidney Foundation; NSAID: Non Steroid Anti-Inflammatory Drug; OI: Opportunistic Infections; OR: Odds Ratio; PLWHA: people living with HIV/AIDS; PRO: Proteinuria; SSA: Sub-Saharan Africa.

## Competing interests

The authors declare that they have no competing interests.

## Authors' contributions

JC, HI, TN and OB contributed to conceptualizing and designing the study. JC, BN, TN, LM, DS, EB, JN participated in the coordination and implementation of the study. BN performed the data collection and EN supervised the data capture. JC and EN performed the data analyses. All authors have been involved in drafting the manuscript.

All authors read and approved the final manuscript.

## Pre-publication history

The pre-publication history for this paper can be accessed here:

http://www.biomedcentral.com/1471-2369/12/40/prepub
